# Yoga maintains Th17/Treg cell homeostasis and reduces the rate of T cell aging in rheumatoid arthritis: a randomized controlled trial

**DOI:** 10.1038/s41598-023-42231-w

**Published:** 2023-09-11

**Authors:** Surabhi Gautam, Romsha Kumar, Uma Kumar, Sanjeev Kumar, Kalpana Luthra, Rima Dada

**Affiliations:** 1 Department of Anatomy, Molecular Reproduction and Genetics Facility, All India Institute of Medical Sciences (AIIMS), New Delhi, India; 2 Present Address: Department of Orthopaedics, Emory Musculoskeletal Institute, Emory University School of Medicine, Atlanta, USA; 3 Department of Biochemistry, All India Institute of Medical Sciences (AIIMS), New Delhi, India; 4 Department of Rheumatology, All India Institute of Medical Sciences (AIIMS), New Delhi, India

**Keywords:** Rheumatoid arthritis, Clinical trial design

## Abstract

The pathogenesis of rheumatoid arthritis (RA) is characterized by a Th17/Treg cell imbalance. A pro-inflammatory cytokine milieu that promotes the continued proliferation of Th17 cells is related to the development of autoinflammation. In RA, T cells have several hallmarks of cellular aging, and they accumulate DNA damage, predisposing to the occurrence of mutations and epigenetic alterations. Since the onset, progression, and treatment response are influenced by a variety of external stressors and environmental factors, this study aimed to evaluate the impact of 8-week yoga practice on disease severity, T cell subsets, markers of T cell ageing and inflammation, epigenetic alterations and gene expression patterns in active RA patients on standard disease-modifying anti-rheumatic drugs (DMARDs). A total of 64 participants with active RA were randomized into 2 groups, yoga group (n = 32) or non-yoga group (n = 32); that were assessed for disease severity, at baseline and after 8 week duration, for Disease Activity Score (DAS28-ESR), T cell subsets [Th17 (CD3+ CD4+ IL17+ RORγt+) cells and Treg (CD3+ CD4+ CD25+ CD127-Foxp3+) cells], markers of T cell aging [aged Th17 cells (CD3+ CD4+ IL17+ RORγt+ CD28−) and aged Treg cells (CD3+ CD4+ CD25+ CD127-Foxp3+ CD28−)], pro-inflammatory markers [IL-6, and IL-17], anti-inflammatory markers [TGF-β, and IL-10], epigenetic alterations [5-methyl cytosine, 5-hydroxymethyl cytosine, and HDAC1] and gene expression patterns [*RORγt, FoxP3, IL-17, IL-6, TGF-*β*, CXCL2, CXCR2,* and *JUN*]. In yoga group, there was a significant improvement in DAS28-ESR scores at the end of 8-weeks of yoga program. The Th17 cells and aged T cell subsets showed a significant decline whereas Treg cell population showed a significant elevation in yoga group. There were significant improvements observed in epigenetic markers as well as inflammatory markers post 8-weeks of yoga practice. The yoga group showed downregulation of *RORγt, IL-17, IL-6, CXCL2, CXCR2,* and upregulation of *FoxP3* and *TGF*-β transcripts. Yoga enables the maintenance of immune-homeostasis as evident by increased Treg cell population and reduced Th17 cell population. Yoga reduces the rate of immunological aging in T cells, as seen by the reduction in population of aged Th17 cells and aged Treg cells. Yoga positively modifies transcriptome and epigenome by normalization of various inflammatory markers, gene expression patterns and epigenetic alterations. Taken together, yoga reduces RA severity, and aids in immune-modulation and hence can be beneficial as an adjunct therapy.

## Introduction

Rheumatoid arthritis (RA) is a chronic inflammatory autoimmune disease of multifactorial origin that develops due to unfavorable coincidence of genetic, immune, and environmental factors^1^. A fragile state of dynamism persists between pro-inflammatory and anti-inflammatory forces. Loss of tolerance to self-antigens is the hallmark of autoimmune disorders leading to the production of autoantibodies^2^. The pathogenesis of RA is heavily influenced by immune cells, particularly B-cells, T-cells, and macrophages^3^. T-cells activate macrophages and fibroblasts to become tissue-destructive cells and release a wide range of cytokines and chemokines to support joint inflammation^4^.

T helper 17 (Th17) and regulatory T cells (Treg cells) are both differentiated from the same naive CD4+ T cells, but in separate cytokine environments and with distinctive gene expression profiles^5^. Pro-inflammatory Th17 cells cause joint injury and autoimmune-derived tissue inflammation by inducing proinflammatory cytokines^6^. Treg cells inhibit the activity of Th17 cells as well as other effector T cells. Treg cells suppress the immune system, maintain self-tolerance, and prevent autoimmune disease by producing anti-inflammatory cytokines^7^. The retinoic acid-related orphan receptor gamma t (RORγt), a lineage-defining transcription factor unique to Th17 cells that produce interleukin (IL)-17, has been linked to a variety of autoimmune diseases^8^. Foxp3 (forkhead transcription factor) plays a crucial role as a lineage specification factor of Treg cells that produce transforming growth factor (TGF)-β, which maintains immune tolerance and homeostasis of the immune system^9^. Th17 cells mediate the pro-inflammatory response through the release of IL-17A and tumor necrosis factor-alpha (TNF-α), which results in tissue destruction as well as damage to bone and cartilage^8^. In contrast, Treg cells mediate the anti-inflammatory response through the release of IL-10 and TGF-β, which aid in maintenance of immune-tolerance^9^. The key immune cells, Treg cells are functionally impaired in RA^10^. The importance of the Th17/Treg cell imbalance in the etiology of RA was highlighted by their opposing behaviors as well as their reciprocal plasticity.

An aberrant Treg cell response with a shift towards a Th17 cell response characterizes the disease onset and course. The relationship between the imbalance of Th17/Treg cells and the production of pro- and anti-inflammatory cytokines is important for the onset and/or course of autoimmunity, chronic inflammation, and articular damage in the joints of RA patients^11^. Age-inappropriate telomere shortening is a hallmark of premature aging in the T cells of RA patients^12^. In RA, the rate of immunosenescence is accelerated^13^. T cells become susceptible to aging as there is enormous proliferative stress due to antigenic exposure, homeostatic proliferation due to thymic involution and their long lifespan as they serve as the carriers of immune memory^14^. The peculiar features of prematurely aged RA T cells include loss of CD28 expression, shrinking naïve and expanding memory repertoire, shortened telomeres, gain of cytotoxicity, DNA damage accumulation, lack an irreversible cell cycle arrest, more vulnerable to apoptosis, altered tissue trafficking, excess pro-inflammatory cytokine secretion^12,15,16^. The inefficient DNA damage sensing and repair machinery of RA T cells are linked to premature T cell aging and arthritogenic effector functions^16^.

Epigenetic alterations like DNA methylation and histone acetylation accumulate with aging and provide a mechanistic link between immunosenescence and development of autoimmune diseases such as RA. T cell landscape is influenced by epigenetic mechanisms such as alteration in the global 5-methyl cytosine (5-mC) DNA, global 5-hydroxymethyl cytosine (5-hmC) DNA and histone deacetylase 1 (HDAC1) levels, which are susceptible to systemic factors, external stressors and environmental stimuli^17^. Yoga is an integrated ancient mind–body practice that is increasingly recognized to have beneficial effects on immune system functioning^18–23^. Yoga practice may exert positive impact on overall health by boosting cell-mediated and mucosal immunity^23^. Our previous studies evaluated the role of yoga as an effective intervention to assist the management of RA with respect to various systemic inflammatory biomarkers, immune-modulatory markers, acute phase reactants, clinical symptoms, depression severity, functional status, pain acuity and quality of life^19,20,24,25^. Yoga aids in regression of inflammatory processes by reducing the pro-inflammatory cytokines and elevating anti-inflammatory cytokines^21^. Nevertheless, the research on exploration of molecular, cellular and epigenetic aspects following yogic practices is rare, hence a possible mode of action underlying the immune-modulatory effects of yoga needs an active exploration. Further investigation is needed to determine how regular yoga practice impacts the T cell subsets that secrete these cytokines, and the associated transcription factors, gene expression and epigenome. There is currently insufficient data to relate yoga's multifaceted therapeutic targets and its molecular mode of action with a malfunctioning immune system as in RA.

Keeping in mind the multifactorial etiology, diverse pathogenesis of RA, heterogeneous clinical phenotypes and the therapeutic potential of yoga, we hypothesized that yoga improves clinical outcome in RA by bringing changes in all interconnected biological components and at various levels—molecular, cellular, organ systems, and the person as a whole. With this novel context in mind, this study aimed to investigate the immune-modulatory effects of 8-weeks of yoga practice on disease severity, T cell sub-sets [Th17 (CD3+ CD4+ IL17+ RORγt+) cells and Treg (CD3+ CD4+ CD25+ CD127-Foxp3+) cells], markers of T cell aging [aged Th17 (CD3+ CD4+ IL17+ RORγt+ CD28−) cells and aged Treg (CD3+ CD4+ CD25+ CD127− Foxp3+ CD28−) cells], inflammatory markers [IL-6, IL-17, TGF-β, and IL-10], epigenetic alterations [5-mC, 5-hmC and HDAC1] and gene expression [*RORγt, FoxP3, IL-17, IL-6, TGF-*β*,* C‐X‐C motif chemokine ligand 2 (*CXCL2),* C‐X‐C motif chemokine receptor 2 (*CXCR2),* and *JUN*].

## Results

### Overview of enrollment

**Figure 1:**
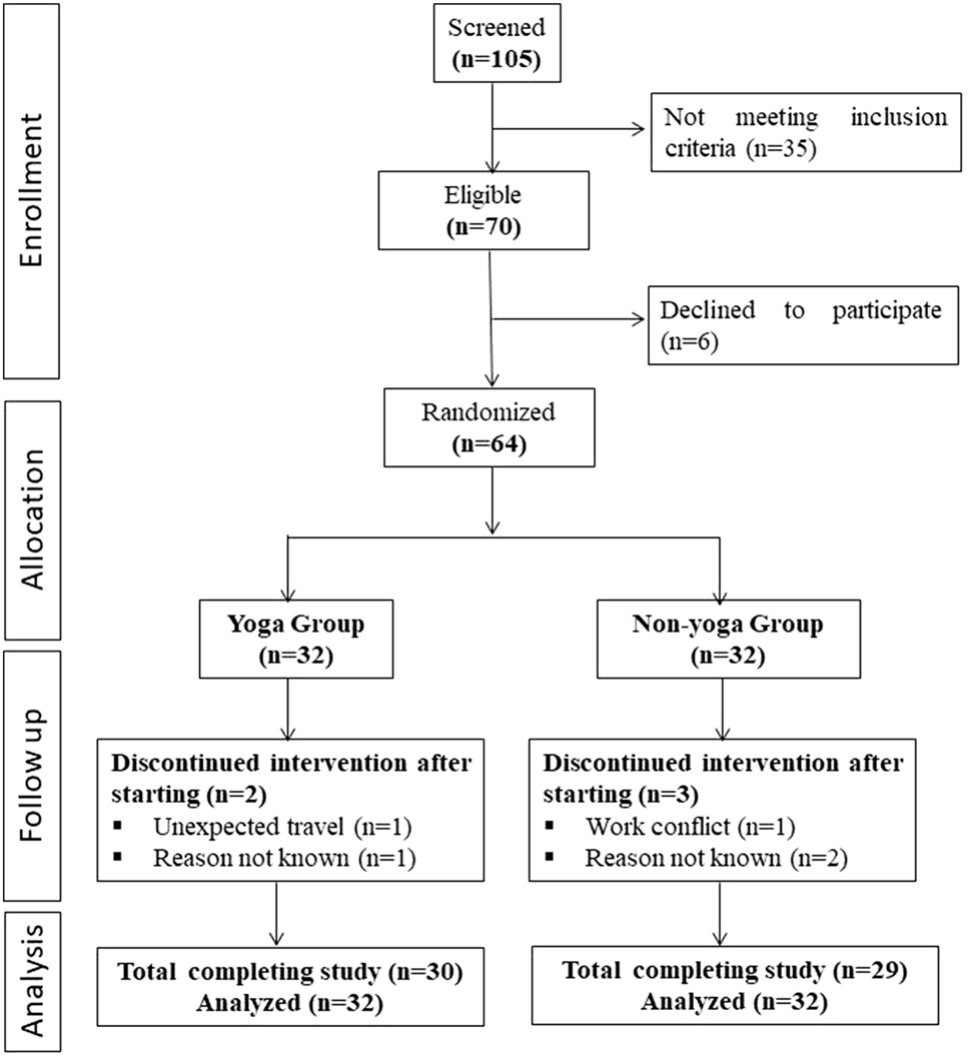
A consort flow diagram of the study.

A total of 105 individuals were screened for eligibility, out of which 64 were randomized into 2 groups (each group *n* = 32). Figure 1 shows the CONSORT flowchart of intervention. All 32 randomized participants were included in intent-to-treat analyses of outcome measures.

### Participants’ baseline characteristics

Baseline demographic and clinical characteristics of all randomized participants are shown in Table 1. There were no statistically significant differences between the two intervention groups, except stratification of disease severity.

**Table 1:** Baseline characteristics. Data were described as frequency (%) for sex, drug therapy, stratification by disease severity, co-morbidity and mean ± SD for others. One asterisk (*) indicates a p-value ≤ 0.05; two asterisks (**) indicate a p-value ≤ 0.01; three asterisks (***) indicates a p-value ≤ 0.001.

Variable	Group (RA patients)		*p* value
	Yoga (n = 32)	Non-yoga (n = 32)	
Demographic characteristics			
Age (years)	46.0 ± 9.4	41.8 ± 9.7	0.0886
Sex			
Male	6	9	0.5561
Female	26	23	
Disease duration (years)	6.6 ± 4.8	5.6 ± 4.4	0.3923
BMI (kg/m^2^)	25.4 ± 4.6	24.0 ± 2.5	0.1303
Presenting symptoms			
Early morning stiffness (minutes)	22.6 ± 22.1	25.5 ± 21.6	0.6092
Tender joint count (TJC)	6.2 ± 4.7	6.7 ± 4.2	0.6557
Swollen joint count (SJC)	4.1 ± 4.3	3.9 ± 2.7	0.7856
Drug therapy			
No. of patients on methotrexate monotherapy	32	32	0.6217
No. of patients on methotrexate plus, other DMARDs	12	9	
No. of patients on biologic response modifiers	0	0	
Disease severity			
Mean DAS28-ESR	4.6 ± 0.9	4.5 ± 1.1	0.4408
Stratification by disease severity			0.0310*
> 2.6–3.2 (low)	1	3	
> 3.2–5.1 (moderate)	14	22	
> 5.1 (high)	17	7	
Co-morbidity			0.9229
DM type 2	8	9	
Hypertension	7	8	
Tuberculosis	6	4	
Hypothyroidism	10	9	
Others	1	2	

### Group × gender interactions

**Figure 2:**
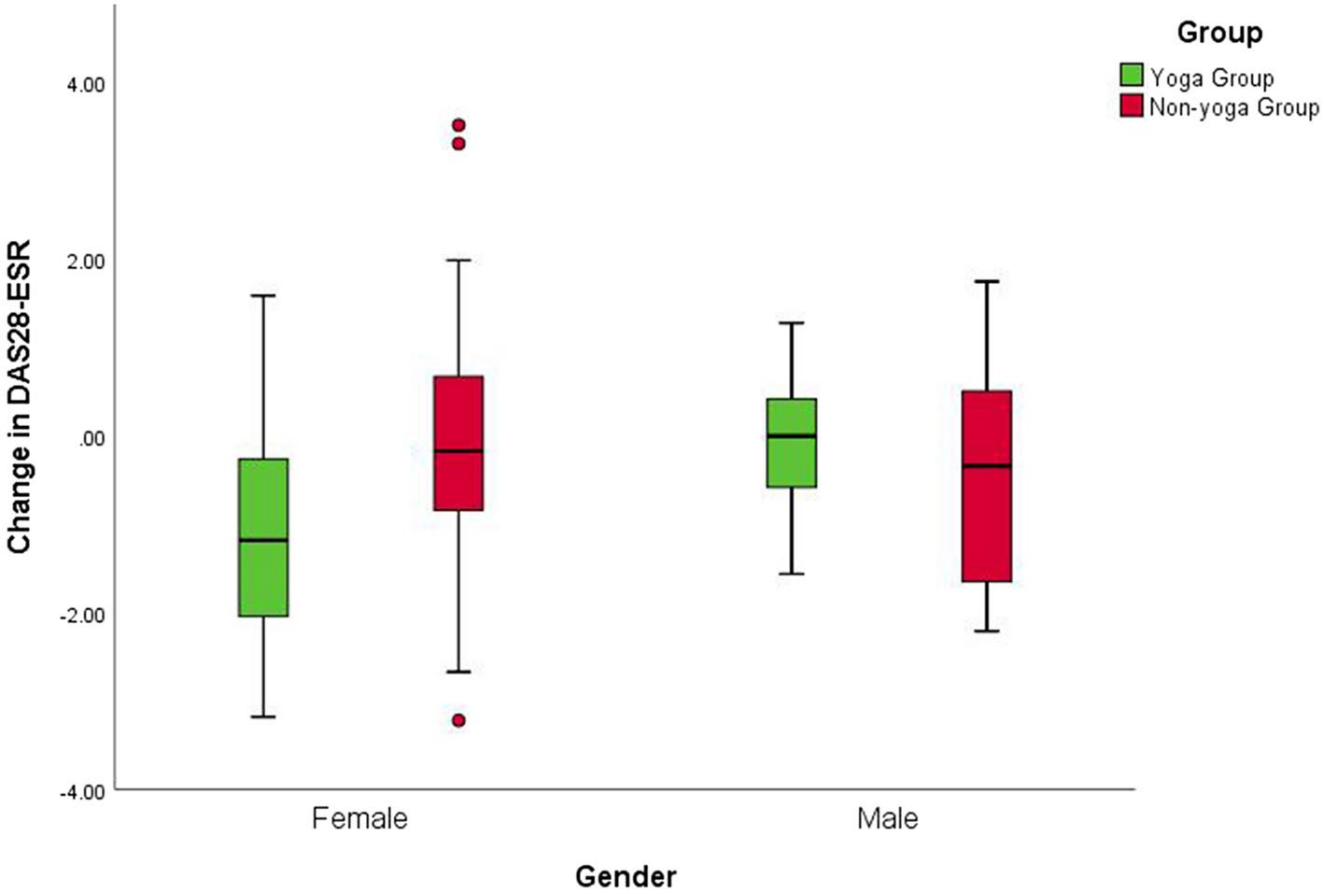
Gender interactions for change in disease activity after the intervention. Mean change of DAS28-ESR score with 95% CI for males in the yoga and the control groups; p = 0.6629 for between-group differences of change in the study, adjusted for baseline value. Mean change of DAS28-ESR score with 95% CI for females in the yoga and the control groups; p = 0.0211* for the between-group difference of change on the study, adjusted for baseline value.

There was no significant difference in mean DAS28-ESR values between males and females at baseline (DAS28-ESR values 4.7** ± **0.9 and 4.6** ± **1.0 mean ± SD, respectively). Separate analyses for males and females were performed to overcome baseline violations in disease activity and to explore further specific gender effects. Interaction effects including group and gender indicated differential responses to yoga for women for DAS28-ESR [F(1,25) = 3.39; p = 0.042], Th17 cells [F(1,25) = 4.06; p = 0.025], Treg cells [F(1,25) = 21.23; p < 0.001], aged Th17 cells [F(1,245 = 14.5; p < 0.001], aged Treg cells [F(1,25) = 13.9; p < 0.001], TGF-β [F(1,25) = 4.62; p = 0.015], 5-mC [F(1,25) = 7.5; p = 0.001] and HDAC1 [F(1,25) = 6.49; p = 0.003]. Clinical improvement was more significant for the women in yoga group [mean between-group difference of change [95% confidence interval (CI)]: female = 1.0, 95% CI (0.15 to 1.8) p = 0.0211; male = −0.27, 95% CI (−1.6 to 1.06) p = 0.6629 (Fig. 2).

### Post-intervention differences in disease activity

There was a significant reduction in DAS28-ESR scores among the participants of yoga group [0.49, 95% (0.13 to 0.85)] with a significant interaction of group and time (ηp^2^** = **4.3; p = 0.042) (Table 2).

**Table 2:** Intent-to-treat analysis: means (SD) and results of within-group and between-group analysis of primary outcomes (*n* = 64; yoga, 32; non-yoga, 32). One asterisk (*) indicates a p-value ≤ 0.05; two asterisks (**) indicate a p-value ≤ 0.01; three asterisks (***) indicates a p-value ≤ 0.001.

Outcome	Yoga group (n = 32)				Non-yoga group (n = 32)			Between groups	Variance analysis/effects				
	Pre		Post	Δ Mean within-group	Pre	Post	Δ Mean within-group	Mean difference (95% CI)^b^	Time		Group × time		
									p^b^ value	η_p_^2^	p^b^ value		η_p_^2^
Disease activity score													
DAS28-ESR	4.7 ± 0.9		3.8 ± 0.9	0.8 ± 1.2	4.5 ± 1.1	4.3 ± 1.3	0.11 ± 1.6	0.49 [0.13 to 0.85]	0.008**	7.4	0.042*		4.3
Inflammatory markers													
IL-6 (pg/ml)	3.4 ± 1.2		2.4 ± 1.6	1.04 ± 1.1	3.7 ± 1.1	3.7 ± 1.4	0.03 ± 1.4	0.54 [0.2 to 0.8]	0.001***	11.9	0.002**		10.4
IL-17 (pg/ml)	191.4 ± 25.8		174.9 ± 25.4	16.4 ± 26.7	187.5 ± 38.9	206.0 ± 41.9	−18.4 ± 21.2	−0.96 [−7.0 to 5.0]	0.750	0.1	< 0.001***		33.4
TGF-β (pg/ml)	40.5 ± 16.9		50.2 ± 16.2	−0.9 ± 13.7	44.8 ± 14.5	43.2 ± 14.5	0.16 ± 11.9	−4.0 [−7.2 to −0.7]	0.015*	6.2	0.001***		12.3
IL-10 (ng/ml)	71.1 ± 12.1		100.9 ± 11.0	−29.7 ± 16.3	66.7 ± 23.4	64.1 ± 24.0	2.5 ± 21.5	−13.5 [−18.3 to −8.8]	< 0.001***	32.2	< 0.001***		45.7
Epigenetic markers													
5-mC (%)		2.2 ± 1.04	3.8 ± 2.9	−1.7 ± 2.8	2.9 ± 1.6	2.6 ± 1.0	0.2 ± 1.4	−0.72 [−1.3 to −0.16]	0.012*	6.6	0.001***		12.8
5-hmC (%)		0.02 ± 0.01	0.007 ± 0.007	0.01 ± 0.02	0.02 ± 0.02	0.01 ± 0.01	0.002 ± 0.02	0.008 [0.003 to 0.014]	0.002**	10.4	0.040*		4.4
HDAC1 (ng/ml)		10.8 ± 7.6	8.3 ± 6.4	2.5 ± 2.9	11.02 ± 6.8	12.2 ± 10.3	−1.1 ± 4.7	0.668 [−0.32 to 1.6]	0.181	1.8	< 0.001***		13.9

### Post intervention differences in molecular markers

#### T cell subsets

**Figure 3:**
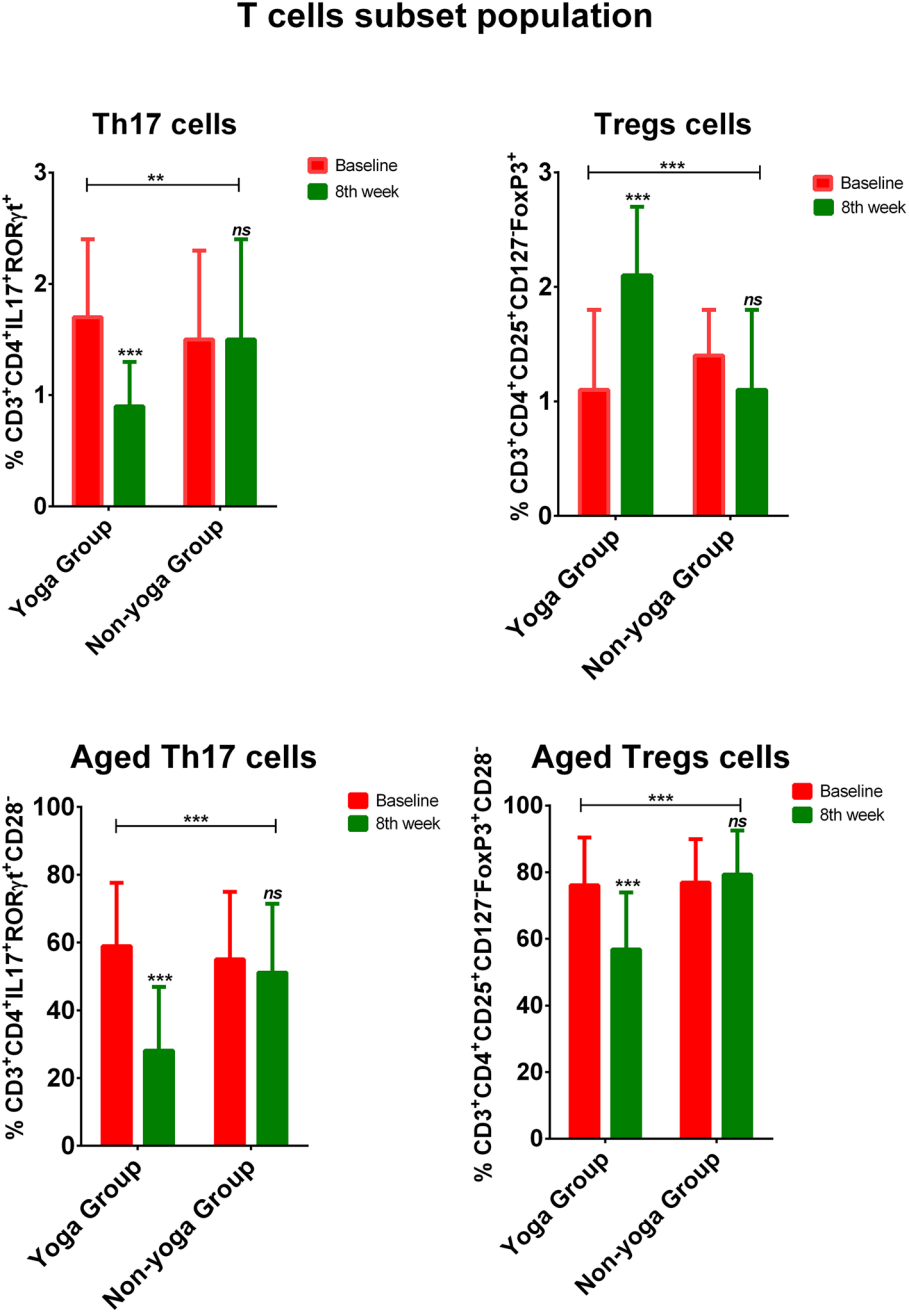
Frequency of Th17, Treg, aged Th17, and aged Treg cells in the yoga group and non-yoga group [p value (ns = p > 0.05; *p ≤ 0.05; **p ≤ 0.01;***p ≤ 0.001)].

The representative graphics from flow cytometry for Th17 and Treg cells measurements are depicted in Supplementary Fig. 1 and 2 respectively. Flow cytometry was performed to evaluate the Th17/Treg cell dynamics and aged T cell populations in both the groups. The mean percentage of Th17 cells (CD3^+^CD4^+^IL17^+^RORγt^+^ T cells) has shown a significant overall decline in yoga group (p = 0.004) from baseline to 8th week [1.7 ± 0.7 vs 0.9 ± 0.4; p < 0.0001], whereas a non-significant difference in non-yoga group from baseline to 8th week [1.5 ± 0.8 vs 1.7 ± 1.1; p = 0.381] (Fig. 3). Conversely, the mean percentage of Treg cells (CD3^+^CD4^+^CD25^+^CD127^-^Foxp3^+^ T cells) has shown a significant overall elevation in yoga group (p < 0.0001) from baseline to 8th week [1.1 ± 0.7 vs 2.1 ± 0.6; p < 0.0001], whereas a non-significant decline in non-yoga group from baseline to 8th week [1.4 ± 0.4 vs 1.1 ± 0.7; p = 0.093] (Fig. 3).

#### Markers of immune aging

A significant overall decline in the mean percentage of aged Th17 cells (CD3^+^CD4^+^IL17^+^RORγt^+^CD28^−^ T cells) was observed in the yoga group (p < 0.0001), from baseline to 8th week [58.9 ± 18.7 vs. 28.1 ± 18.8; p < 0.0001], whereas a non-significant difference in the non-yoga group from baseline to 8th week [55.0 ± 19.9 vs. 51.1 ± 20.3; p = 0.406] (Fig. 3). Similarly, a significant overall decline in the mean percentage of aged Treg cells (CD3^+^CD4^+^CD25^+^CD127^−^Foxp3^+^CD28^−^ T cells) was seen in the yoga group (p < 0.0001) from baseline to 8th week [76.1 ± 14.3 vs. 56.8 ± 17.1; p < 0.0001], with no significant alterations observed in the non-yoga group from baseline to 8th week [76.9 ± 13.0 vs. 79.3 ± 13.2; p = 0.329] (Fig. 3).

#### Inflammatory markers

There were significant changes observed in various inflammatory markers after 8-weeks of intervention in yoga group as compared to non-yoga group. Pro-inflammatory cytokines like IL-6 [0.54, 95% CI (0.22 to 0.85); ηp^2^ = 10.4; p = 0.002], IL-17 [−0.96, 95% CI (−7.0 to 5.06); ηp^2^ = 33.4; p < 0.001] showed a significant decline with respect to interaction of group and time; whereas anti-inflammatory cytokines like TGF-β [−0.4, 95% CI (−0.7 to −0.07); ηp^2^ = 12.3; p = 0.001] and IL-10 [−13.5, 95% CI (−18.3 to −8.8); ηp^2^ = 45.7; p < 0.001] showed a significant increase in yoga group as compared to control group with respect to interaction of group and time (Table 2). Also, the change in mean within non-yoga group showed significantly increased levels of IL-17 [−18.4 ± 21.2; p < 0.001] compared to baseline levels (Table 2).

#### Epigenetic alterations

The percentage of global 5-mC was significantly higher in the yoga group as compared to the non-yoga group [−0.72, 95% CI (−1.3 to −0.16); ηp^2^ = 12.8; p = 0.001] (Table 2). Conversely, the percentage of global 5-hmC was significantly reduction in the yoga group after 8-weeks of intervention with a significant mean difference observed by group analysis [0.008, 95% CI (0.003 to 0.014); ηp^2^ = 4.4; p = 0.04)] (Table 2). Also, the HDAC1 levels were found to be significantly reduced after 8-weeks of intervention in the yoga group as compared to the non-yoga group [0.668, 95% CI (−0.32 to 1.6); ηp^2^ = 13.9; p < 0.001)] (Table 2).

#### Gene expression levels

**Figure 4:**
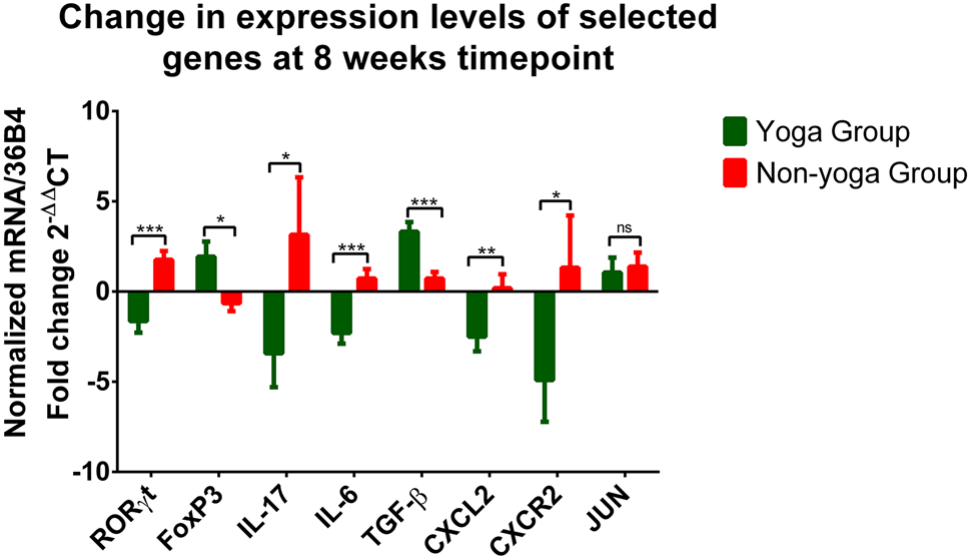
The relative mRNA expression levels of dysregulated transcripts in the yoga group and non-yoga group [p value (ns = p > 0.05; *p ≤ 0.05; **p ≤ 0.01;***p ≤ 0.001)].

The results for expression analysis showed significant downregulation in relative mRNA expression levels of *RORγt* (p = 0.0002)*, IL-17* (p = 0.0219)*, IL-6* (p = 0.0003)*, CXCL2* (p = 0.0065)*,* and *CXCR2* (p = 0.0284) in the yoga group as compared to the non-yoga group. The mean axis fold change of these transcripts was as follows in the yoga vs. non-yoga group: *RORγt* (−1.64 vs. 1.74), *IL-17* (−3.42 vs. 3.15), *IL-6* (−2.34 vs. 0.72), *CXCL2* (−2.50 vs. 0.19), and *CXCR2* (−4.90 vs. 1.32) respectively. The mRNA expression levels of *JUN* were not found to be different statistically (p = 0.7237) with a mean axis fold change of 1.05 vs. 1.38 in the yoga vs. non-yoga group. In the yoga group, the mRNA expression levels were significantly upregulated with the mean axis fold change of *FoxP3* (1.93 vs. −0.65; p = 0.0169) and *TGF-*β (3.32 vs. 0.72; p = 0.0001) in the yoga vs. non-yoga group respectively (Fig. 4).

## Discussion

Our study is the first to highlight the positive impact of yoga on modulation of the T cell subsets, T cell aging markers, epigenetic alterations and associated transcription factors in RA. We found that 8 weeks of yoga practice significantly reduced disease activity, normalized the biomarkers associated with inflammation, and maintained Th17/Treg cell homeostasis. Further, yoga reduced the rate of immunological aging as seen by the reduction in the aged Th17 cell population (CD3+ CD4+ IL17+ RORγt+ CD28-T cells) and aged Treg cell population (CD3+ CD4+ CD25+ CD127-Foxp3+ CD28-T cells). Our findings suggest that yoga positively modified epigenetic changes such as global methylation levels, global hydroxyl methylation levels, and HDAC1 levels which may regulate gene expression patterns. The yoga group showed the downregulation of *RORγt, IL-17, IL-6, CXCL2, CXCR2,* and upregulation of *FoxP3* and *TGF*-β. These findings suggest that yoga possesses an immune-modulatory potential which induces molecular remission in RA by influencing its pathobiology at both cellular and molecular level.

In the present study, the impact of regular practice of yoga was beneficial as there was a significant reduction in the systemic levels of pro-inflammatory markers (IL-6 and IL-17) and various transcripts associated with pro-inflammatory cytokines. IL-6 is essential for the development of RA's systemic(lung, heart skin, brain) and joint inflammation, immune system abnormalities, and joint swelling^30^. The systemic inflammatory signs and symptoms of RA, which are mediated by IL-6, include fever, malaise, sleep disturbances, muscular weakness, and anemia^31^. In order to promote the production of adhesion molecules and draw leukocytes to the affected joints, IL-6 induces endothelial cells to secrete IL-8 and monocyte chemoattractant protein-1 (MCP-1) locally at joint level^32^. IL-6 can promote osteoclast differentiation and synoviocyte proliferation by activation of the NF-kappa B receptor activator ligand (RANKL). Through STAT3-mediated activation of the ROR*γ*t in the presence of TGF-β, IL-6 stimulates the differentiation of Th17 cells while inhibiting TGF-induced Treg cell differentiation^33^. Thus, IL-6 encourages Th17 over Treg dominance in the effector CD4+ T cell subsets, which is regarded to be a key factor in the emergence of RA and other immune-mediated illnesses^30^. Another important inflammatory marker, IL-17 affects RA joints by promoting the development of matrix metalloproteases and pro-inflammatory cytokines, which contribute to tissue inflammation and destruction^34^. Th17 cells play a unique role in immune activity through the generation of effector cytokines such as IL-17A, IL-17F, IL-22, etc. Th17 cells influence the development of osteoclasts and bone resorption by the release of IL-17, an activate monocytes to create pro-inflammatory cytokines, accelerating the inflammatory cascade^35,36^. Expression of CXCL2 was significantly increased in RA patients and was associated with bony erosions^37^. CXCL2 is a pro-inflammatory factor which is associated with biological signs of progress, such as angiogenesis, inflammation and cancer. CXCL2 stimulated osteoclastogenesis via extracellular receptor kinase (ERK) mitogen‐activated protein kinase (MAPK) and nuclear factor kappa B pathways^37^. Leukocyte trafficking is regulated by chemokines both during homeostasis and immunological responses. CXCR2 plays a cell-autonomous function in the migration of neutrophils to inflamed joints, which is essential for the emergence of auto-immune arthritis^38^. Furthermore. our study demonstrates that yoga practice down-regulates the mRNA transcript levels of pro-inflammatory genes (*RORγt, IL-6, IL-17, CXCL2, CXCR2*), hence reducing systemic and local inflammation in RA, thus overall providing a clear clinical and molecular evidence of the impact of yoga on RA. Yoga, is mind body energy medicine, comprises of both psychological and physical components and is thus ideal as an adjunct management system of this severe painful disabling autoimmune arthritis like RA and has been documented from previous studies from our group for its role as a powerful adjunct to modern medicine in disease management, rehabilitation and promotion of health.

Yoga is a centuries-old method of unifying the mind, body, and soul. The Patanjali’s ashtanga (eight limb) Yoga includes yama (abstinences), niyama (observances), asana (yoga postures), pranayama (breath control), pratyahara (withdrawal of the senses), dharana (concentration), dhyana (meditation) and samadhi (absorption)^18^. Dharna, dhyana and samadhi are *antaranga* yoga. Yoga originated from the belief that stress is the cause of all illnesses, which gives the practice advantages for both physical and mental health^39^. Stress impacts every cell of the body and every organ system and yoga thus targets both the mind and body reducing stress by building emotional resilience by promoting neuroplasticity. Not only is there increase in several neurochemicals like brain derived neurotrophic factor (BDNF), dehydroepiandrosterone (DHEA), melatonin, serotonin but also actual anatomical changes in the brain in the hippocampus, prefrontal cortex which show increase in gray matter and concomitant decrease in size of amygdala. IL-10 possesses anti-inflammatory and immunoregulatory properties. Yoga has the potential to mediate the inflammatory pathways by increasing the levels of anti-inflammatory mediators and reducing the pro-inflammatory markers^19,40^. IL-10 prevents T-cell responses to specified antigens and inhibits the synthesis of proinflammatory cytokines and chemokines^41^. The main mechanism by which IL-10 exerts its effects is by inhibiting the costimulatory capabilities of macrophages and promoting the proliferation and differentiation of antibody-forming B cells. The effectiveness of IL-10 in reducing inflammation and autoreactivity has been demonstrated in preclinical research using a range of animal models, including collagen-induced arthritis^42^. TGF-β is a regulatory cytokine which promotes the growth of Treg cells and controls CD4+ T cell polarization. Depending on the cytokine environment, TGF- β stimulates the differentiation of Th17 and Treg cells^43^. The transcription factor ROR*γt*, which is the principal regulator of Th17 cells, is activated and controlled in its expression by TGF- signaling and STAT3^44^. TGF-β can induce the differentiation of CD4+ T cells into Th17 cells in the presence of IL-6 or IL-21. Our results have shown that the yoga group showed significant upregulation of mRNA expression levels of *FoxP3* and *TGF-*β as compared to the non-yoga group, further confirming the immune-modulatory role of yoga. The dimers of members of the Jun, Fos and activating transcription factor protein families make up the transcription factor activator protein 1 (AP-1)^45^. Jun mostly acts as a proliferating growth regulator of cells^46^.In the present study, the mRNA expression levels of *JUN* had shown a declining trend in the yoga group; however, the difference was not statistically significant as compared to the non-yoga group.

The immune dysregulation in RA is attributed to increased secretion of inflammatory cytokines by effector Th17 cells and a loss of Treg cells suppressor function. Yoga's anti-inflammatory properties work to restore immune homeostasis to its ideal state and promote natural immunological tolerance to treat autoimmune diseases^20,21^. Our results also showed that regular practice of yoga was associated with increased Treg cell population and decreased Th17 cell population. Our findings imply that yoga-induced variations in serum cytokine levels stimulate changes in Treg and Th17 cell populations, which may shift the delicate immune system balance to reestablish tolerance. A study on the effect of a 12-week program of regular tai chi chuan exercise showed beneficial effects on functional mobility, beliefs about the benefits of exercise on physical and psychological health and maintained immune regulation in middle-aged volunteers by increase in their Treg cells^47,48^. Our results were in concordance with a study conducted on the ischemic cardiomyopathy rat model in which the Th17/Treg ratio was much lower in the rats trained on treadmill (Slope 0°, 12 m/min, 30 min/time) five times per week, for 12 weeks than non-training group rats^49^.Few more studies focused on the impact of endurance training and physical exercise on athletes where a diversion in the Th17/Treg cell balance was seen^49,50^. Our study has documented a post-yoga reduction in the mean percentage of Th17 cells (CD3+ CD4+ IL17+ RORγt+ T cells) in yoga group from baseline to 8th week whereas the mean percentage of Treg cells (CD3+ CD4+ CD25+ CD127− Foxp3+ T cells) has shown a significant elevation in yoga group. RA patients experience the signs of accelerated ageing like reduced thymic functioning, expansion of late-differentiated effector T cells, increased telomeric attrition, and excessive production of cytokines (senescence-associated secretory phenotype)^12^. Early onset of age-related co-morbidities like osteoporosis, cardiovascular issues, and cognitive decline have all been linked to the progression of RA^51^. The crucial co-stimulatory protein CD28 is expressed on undifferentiated CD4+ and CD8+ T lymphocytes. Since the MHC-I-bound peptide antigen has a low affinity, activating T cells using antigen-presenting cells (APCs) alone is not enough to fully activate and sustain T cells^52^. To extend T cell responses, the co-stimulatory signal from the CD28 cell surface receptor that binds with CD86 or CD80 on antigen presenting cells is required. Yoga possesses the potential to reduce the pace of immunological aging which can be attributed to the reduced mean percentage of aged Th17 cells (CD3+ CD4+ IL17+ RORγt+ CD28-T cells) as well as aged Treg cells (CD3+ CD4+ CD25+ CD127− Foxp3+ CD28-T cells) after 8 weeks of practice.

Inflammatory changes may be influenced by epigenetic mechanisms that subsequently lead to gene expression alterations and, eventually, protein expression^53^. The epigenome controls gene expression and is susceptible to changes brought on by stress, lifestyle changes and other environmental variables^54^. The most extensively researched epigenetic modification is DNA methylation, which is increasingly recognized as a meaningful biomarker in the pathogenesis of RA^55^. High levels of inflammation and poor physical health have both been linked to changes in DNA methylation^56^. Since epigenetic modifications have the capacity to be reversed, they can be used to gauge how well clinical treatments are working. Recent studies have reported a global DNA hypomethylation in immune cells of RA patients as compared to healthy individuals^57^. Global DNA hypomethylation is implicated in various inflammatory and autoimmune disorders and leads to aberrant gene expression^58^. In RA, the low levels of methylation at specific CpG sites in fibroblast-like synoviocytes is correlated with overexpression of genes which are important for the disease pathogenesis such as growth factors/ receptors, extracellular matrix proteins, adhesion molecules, and matrix degrading enzymes, etc^59,60^. In our study, we have seen an overall increase in the percentage of global 5-mC DNA in yoga group over non-yoga group after 8-weeks of yoga intervention, which might lead to the downregulation of pro-inflammatory genes like *RORγt, IL-6, IL-17, CXCL2, CXCR2,* etc. DNA demethylation also occurs via oxidative modification followed by removal of the base^61^. Active DNA demethylation occurs due to the presence of 5-hmC. In order to study the effect of yoga on the status of DNA demethylation in RA patients, the global 5hmC percentage was estimated. This is the first study to document an overall decrease in the percentage of global 5-hmC DNA in yoga group over non-yoga group after 8- weeks of yoga intervention. Modifications of histones are major epigenetic markers that regulate gene expression and establish various cell phenotypes^62^. The open structure of chromatin makes the chromatin available to transcription factors and can increase gene expression dramatically. Histone acetylation is important for gene expression regulation, while histone deacetylation contributes to chromatin condensation and gene transcription repression^63^. Inhibition of HDAC function by epigenetic or non-epigenetic mechanisms can lead to the development of RA and other autoimmune inflammatory diseases, affecting the complex modulation of intracellular signaling pathways^64^. HDAC inhibitors exhibit anti-arthritic activities through modulation of only a small percentage of gene expression involved in chronic inflammation and apoptosis^65^. HDAC inhibitors act via epigenetic and non-epigenetic processes to generate immunomodulatory effects. HDAC inhibitors demonstrated anti-inflammatory effects in animal models of arthritis and synovial tissues in RA patients^66^. In our study, we have seen a reduction in the levels of HDAC1 in yoga group as compared to non-yoga group after 8-weeks of yoga. The effects of HDAC inhibitors on cellular activation have been attributed to epigenetic regulation, signal transduction modifications, and gene expression modulation via regulation of mRNA stability.

Lack of an active control group was one of the study's shortcomings because the non-yoga group had no equal attention control intervention and only received medication therapy in comparison to the active yoga intervention group. Therefore, including such a group would further rule out the therapeutic results that were specifically linked to the yoga intervention. In both the yoga and non-yoga groups, there were fewer men, which was explained by the fact that women had a higher prevalence of RA than men (3:1). It is challenging to maintain a regular schedule for a long-term RA management or home practice regimen because each session of yoga lasted for 120 min every day while being supervised by a certified yoga instructor. Also, the lack of long-term follow-up of the participants made it difficult to predict how quickly participants returned to their baseline levels. In order to investigate the long-term advantages of the yoga practice, we intend to conduct additional research with a large sample size and long-term follow-ups and practice of yoga for shorter duration.

In conclusion, our study findings highlight that yoga possesses an immune-modulatory potential which induces molecular remission and reestablishes immunological tolerance in RA by influencing its pathobiology by optimizing inflammatory markers, maintaining immune-homeostasis, reducing the rate of immune-aging and improving RA health outcome. The 8 weeks of yoga practice significantly reduces disease activity, maintains Th17/Treg cell homeostasis and reduces inflammatory processes by optimizing the levels of various pro-inflammatory cytokines, and anti-inflammatory cytokines with changes in gene expression patterns. Yoga positively modifies the epigenome by elevating global methylation levels, reducing global hydroxyl methylation levels, and HDAC levels which may cause the normalization of dysregulated gene expression. Hence, yoga can be used as an adjuvant therapy in RA as it boosts physical functioning, enhances psychological wellbeing and reestablishes immunological tolerance.

## Methods

### Ethics declarations and study design

This study was a prospective, single-blinded, randomized controlled trial with active RA patients, aimed at analyzing the effects of an 8-week yoga practice in RA patients on standard drug therapy. The study was initiated after obtaining ethical clearance (IECPG-211/24.02.2016) from the Institute Ethics Committee of AIIMS, New Delhi, India, and registration under the clinical trials registry, India (CTRI/2017/05/008589, Registered on 17.05.2017). All methods were performed in accordance with the relevant guidelines and regulations. All the participants gave written informed consent before the study protocol's commencement.

### Participants and eligibility criteria

The study participants were recruited from the outpatient unit of Rheumatology department of AIIMS, New Delhi. RA patients, 18–60 years old, diagnosed as per 2010 ACR/EULAR RA classification criteria^26^, whose DAS28ESR was > 2.6 and who were on standard medical treatment for at least 6 months were recruited after obtaining written informed consent. Patients with any other overlapping autoimmune diseases, chronic systemic diseases, history of administration of oral or intra-articular steroids in the previous six months, medical treatment for any other illnesses, intake of any form of herbo-mineral, antioxidant, homeopathic, or ayurvedic supplementation (*Boswellia serrata*, *Ricinus communis,* dry ginger powder, fenugreek seeds, curcumin, *ashwagandha* etc.) or already practicing yoga and meditation in any way were excluded from the study. Also, during the execution of the study, the events which resulted in the exclusion were the inability to comply with regular yoga and deviating from their usual lifestyle habit.

### Sample size calculation

The sample size calculation for the study assumed to detect a standardized effect size [difference in mean change in DAS28-ESR between the two groups/pooled standard deviation (SD)] of 0.8 with a 95% confidence level and 80% power, considering the mean and SD of a previous study by Evans et al. (2011)^27^. Considering some loss to follow-up, we enrolled a total of 64 patients in the study and randomized into two groups—yoga group (32 patients) and non-yoga group (32 patients).

### Randomization

For this investigator blinded study, sequentially labeled sealed opaque envelopes were used to conceal random numbers as described earlier^20^. As each patient was enrolled, they were assigned to the next numbered envelope and the associated group, either yoga group or non-yoga group. The sequence of random numbers was generated by permuted block randomization of variable block size with the assistance of the web tool research randomizer (https://www.randomizer.org/). The participants were not blinded to the study, whereas investigators who interviewed patients, conducted experiments, and performed statistical analyses blinded to the group status of the patients.

### Intervention

The participants were randomized into two groups- the yoga group and non-yoga group. All the study participants were asked to undergo a clinical evaluation and provide a blood sample on day 0 (baseline) and the end of 8^th^ week of intervention.

#### Yoga program (yoga group)

As described previously^19^, the participants of the yoga group were administered a standardized yoga program for eight weeks, which was suitable for active RA patients, so that it did not cause any further irritation to already inflamed joints. The patients with deformed joints and limitations were advised to undergo customized physical postures, relaxation exercises, and practiced breathing forming a slow and deep breathing pattern with exhalation being longer than inhalation. The comprehensive yoga program incorporated the components of Patanjali's ashtanga yoga. Briefly, the intervention comprised of yogic practices including asanas (physical postures), pranayama (regulated breathing techniques), dhyana (meditation), and savasana (relaxation techniques), followed by interactive counseling sessions on yoga, stress management, nutrition, as well as personal lifestyle management (Supplementary Fig. 3). Yoga program was administered five times a week for 120 min duration per session for 8 weeks^19,25^. The sessions were conducted by the registered and well-qualified yoga instructor at the Laboratory for Molecular Reproduction & Genetics, Department of Anatomy, AIIMS, New Delhi. Adherence was monitored with participant diary and yoga teacher's remarks at each visit. As such, there was no home regimen to be followed for the yoga practice due to the extensive time commitment of each session. The patients were encouraged to incorporate yoga into their lifestyle after the end of the 8 weeks of intervention. The patients of this group continued with their standard disease-modifying anti-rheumatic drugs (DMARDs) as per the prescription of rheumatologists.

#### Usual care control (non-yoga group)

As described previously^19^, the patients assigned to the non-yoga group continued with their ongoing medical care, which included DMARDs prescribed by the rheumatologists. The patients followed their normal daily physical activities with no change in their daily lifestyle for eight weeks.

### Outcome measures

#### Primary outcome

The disease activity of the patients was assessed by DAS28-ESR^28^. The DAS28-ESR consisted of four components: tender joint count, swollen joint count, visual analog scale (VAS) score of the patient’s global health, and ESR. In DAS28-ESR, a rating of ≤ 2.6 represents remission, > 2.6 to 3.2 represents low disease activity, > 3.2 to 5.1 represents moderate disease activity, and ≥ 5.1 represents high disease activity.

Change in disease severity was measured by disease activity score—erythrocyte sedimentation rate (DAS28-ESR) from baseline (day 0) to 8-weeks and recorded as primary outcome.

#### Secondary outcome

All parameters were evaluated on day 0 (baseline) and 8th week (follow-up) of the intervention and the fasting blood samples were obtained at 8 am in the morning.

Alterations in cellular and molecular markers including: (a) T cell subset population: Th17 (CD3+ CD4+ IL17+ RORγt+) cells and Treg (CD3+ CD4+ CD25+ CD127− Foxp3+) cells, (b) markers of immune aging: aged Th17 (CD3+ CD4+ IL17+ RORγt+ CD28−) cells and aged Treg (CD3+ CD4+ CD25+ CD127− Foxp3+ CD28−) cells, (c) inflammatory markers: IL-6, IL-17, TGF-β, and IL-10 (d) epigenetic alterations: 5-methyl cytosine, 5-hydroxymethyl cytosine and HDAC1, and (e) gene expression levels: *RORγt, FoxP3, IL-17, IL-6, TGF-*β*,CXCL2,CXCR2,* and *JUN* were documented.

### Measurement of molecular markers

Following techniques were employed for the measurement of molecular markers:

#### Phenotyping of T cell subsets by flow cytometry

The isolated PBMCs (1 × 10^6^ cells /ml) were stained with a panel of fluorochrome-conjugated monoclonal antibodies (Thermo Fisher Scientific, USA) for the surface markers, namely CD3, CD4, CD28, CD25, and CD127 and intra-cellular cytokines/transcription factors, namely IL-17, RORγt, and FoxP3. Alexa Fluor 488-labeled anti-human CD3, PerCP Cy5.5-labeled anti-human CD4, APC- labeled anti-human IL-17, PE-labeled anti-human RORγt were used to examine CD3^+^CD4^+^IL17^+^RORγt^+^ T cells. Alexa Fluor 488-labeled anti-human CD3, PerCP Cy5.5-labeled anti-human CD4, PE-labeled anti-human CD25, APC-eFluor 780-labeled anti-human CD127, and APC-labeled anti-human FoxP3 were used to detect CD3^+^CD4^+^CD25^+^CD127^−^Foxp3^+^ T cells. PE-Cyanine7-labeled anti-human CD28 was used for aged T cell population i.e. aged Th17 cells(CD3^+^CD4^+^IL17^+^RORγt^+^CD28^−^ T cells) and aged Treg cells (CD3^+^CD4^+^CD25^+^CD127^-^Foxp3^+^CD28^−^ T cells).

The isolated PBMCs (1 × 10^6^ cells /ml) were taken in FACS tubes for immuno-staining. The recommended value of monoclonal antibodies (for surface markers as mentioned above) were added, mixed thoroughly, and incubated on ice or 4 °C for 45 min in the dark. Cells were washed with staining buffer (1× PBS with 1% FBS) and pelleted at 1200 rpm for 10 min. For intracellular staining, cells were permeabilized and added with permeabilization buffer (Thermo Fisher Scientific, USA). The cell pellet was resuspended in 100 μl of transcription factor staining buffer (Thermo Fisher Scientific, USA), and with the recommended value of monoclonal antibodies (for transcription factors & intracellular cytokines) and incubated in the dark at room temperature for 1 h. After two steps of washing with PBS, the cells were resuspended in 100 μl of with permeabilization buffer (Thermo Fisher Scientific, USA) and fixed by adding 20 μl of 4% paraformaldehyde (PFA) and stored at 4 °C. Fixed cells were flow- cytometrically acquired & analyzed by BD Fortessa X20 flow cytometer (BD Biosciences, San Jose, CA) equipped with FACSDIVA™ software. Fifty thousand events were acquired per sample within a typical forward, and side scatter gate set to exclude dead cells and debris. Data were analyzed using FlowJo 7.22 (Tree Star Ashland, Orlando, FLA) software.

#### Detection of inflammatory and epigenetic markers

Serum levels of IL-6 (Gen-Probe, Diaclone Diagnostic, France), IL-17 (Gen-Asia Biotech, China), IL-10 (Bioassay Tech Laboratory, China), and HDAC1 (Qayee Bio-Technology) were estimated by ELISA using commercially available kits. Briefly, the workflow of the HDAC1 ELISA kit utilizes the sandwich ELISA methodology where the plate was pre-coated with human HDAC1 antibody. The serum samples were then added onto the pre-coated wells. And, then biotinylated human HDAC1 antibody was added, which binded to HDAC1 in the sample. After the addition of Streptavidin-HRP developer followed by substrate and stop solution, the well absorbance for HDAC1 was measured at 450 nm. The 5-methylcytosine DNA ELISA kit (Enzo Life Sciences, Inc., USA) and 5-hydroxymethyl cytosine DNA ELISA kit (Enzo Life Sciences, Inc., USA) was used to quantify the percent 5-mC DNA and 5-hmC DNA respectively. Briefly, the workflow for the 5-mC DNA ELISA kits utilize the indirect ELISA methodology where 100 ng denatured, single-stranded DNA (ssDNA) samples per well were coated on the plate wells and a 5-mC mAb and conjugate HRP-Ab were then added to the wells. The detection of 5-mC was done after the addition of the HRP developer by measuring well absorbance at 405–450 nm. Briefly, the workflow of the 5-hmC DNA ELISA kit utilizes the sandwich ELISA methodology where a 5-hmC pAb was coated to the bottom of plate well surfaces. The ssDNA (100 ng/well) sample was then added onto the well surface which binded to 5-hmC pAb’s and was then recognized by a conjugate DNA HRP-Ab. After the addition of HRP developer, the well absorbance for 5-hmC DNA was measured at 405–450 nm. Serum TGF-β levels were estimated by a magnetic bead-based multiplex assay using Bio-Plex Pro TGF-β Assays (Bio-Rad Laboratories Inc., USA) according to the manufacturer's guidelines. Quality-control assays for biomarkers and validation were performed.

#### Detection of gene expression patterns

As described earlier^20^, total RNA was isolated from 1 ml of freshly obtained EDTA blood by TRIzol manual method followed by complementary DNA (cDNA) synthesis by reverse transcribing 1000 ng of RNA by using the iScript cDNA synthesis kit (BioRad). The CFX96 realtime system (BioRad, CA, United States) quantified the relative gene expression using Brilliant III UltraFast SYBR Green qPCR Master Mix. The protocol for gene amplification was standardized at 35 cycles. Normalization of the amount of expressed mRNA used two internal housekeeping genes *36B4* and *β-actin*. The relative fold of gene expression was done by the 2^−ΔΔCt^ method^29^. The primer sequences for the genes used in the study are shown in Table 3.

**Table 3:** List of primer sequences.

S. no.	Gene	Primer sequence (5′ to 3′)
1	*RORγt*	Forward: CCTGGGCTCCTCGCCTGACC Reverse: TCTCTCTGCCCTCAGCCTTGCC
2	*FoxP3*	Forward: GTGGCCCGGATGTGAGAAG Reverse: GGAGCCCTTGTCGGATGATG
3	*IL-17*	Forward: CGGCTGGAGAAGATACTGGT Reverse: TTAGTCCGAAATGAGGCTGTC
4	*IL-6*	Forward: GGCACTGGCAGAAAACAACC Reverse: GCAAGTCTCCTCATTGAATCC
5	*TGF-*β	Forward: GAAGGGAGACAATCGCTTTAGC Reverse: TGTAGACTCCTTCCCGGTTGAG
6	*CXCL2*	Forward: TGCCAGTGCTTGCAGAC Reverse: TCTTAACCATGGGCGATGC
7	*CXCR2*	Forward: TGGCTTGATCAGCAAGGACTC Reverse: GCCCTGAAGAAGAGCCAACA
8	*JUN*	Forward: CAGCTTCATGCCTTTGTA Reverse: CTCAGAGTGCTCCAAATCTC
9	*36B4*	Forward: AACATGCTCAACATCTCCCC Reverse: CCGACTCCTCCGACTCTTC
10	β-*actin*	Forward: TGAGAGGGAAATCGTGCGTG Reverse: TGCTTGCTGATCCACATCTGC

### Statistical analysis

All statistical analyses were carried out on an intent-to-treat basis with the baseline observation carried forward approach using IBM SPSS Statistics for Macintosh, Version 25.0. (IBM Corp. Armonk, NY, United States) and GraphPad Prism Version 6.01. (GraphPad Software, Inc., San Diego, CA). Chi-square test and Fisher’s exact test compared the baseline characteristics between the two groups. The assessment of interaction effects among baseline parameters was carried out by mixed factorial design ANOVA. For within-group analysis, paired t-test was used to study the difference between pre- to post-intervention for normally distributed data, or Wilcoxon signed-rank tests for continuous variables without normal distribution. For between-group analysis, the repeated measure ANOVA was used to study the intervention effects along with the interaction of time and group. A *p-*value of < 0.05 was considered to be statistically significant.

### Supplementary Information

Supplementary Figures.

## Data Availability

The datasets used and/or analyzed during the current study available from the corresponding author on reasonable request.
