# Supplementary material for: Yoga maintains Th17/Treg cell homeostasis and reduces the rate of T cell aging in rheumatoid arthritis: a randomized controlled trial
Source: Sci Rep. 2023 Sep 11;13:14924. doi: 10.1038/s41598-023-42231-w (PMC10495372; doi:10.1038/s41598-023-42231-w)
Supplement: Supplementary file 1 — Supplementary Figures. [file 41598_2023_42231_MOESM1_ESM.pdf]

SUPPLEMENTARY MATERIAL

Yoga maintains Th17/Treg cell homeostasis and reduces the rate of T cell aging in Rheumatoid arthritis: A randomized controlled trial

Surabhi Gautam[1], Romsha Kumar[2], Uma Kumar[3], Sanjeev Kumar[2], Kalpana Luthra[2], Rima Dada[1,*]

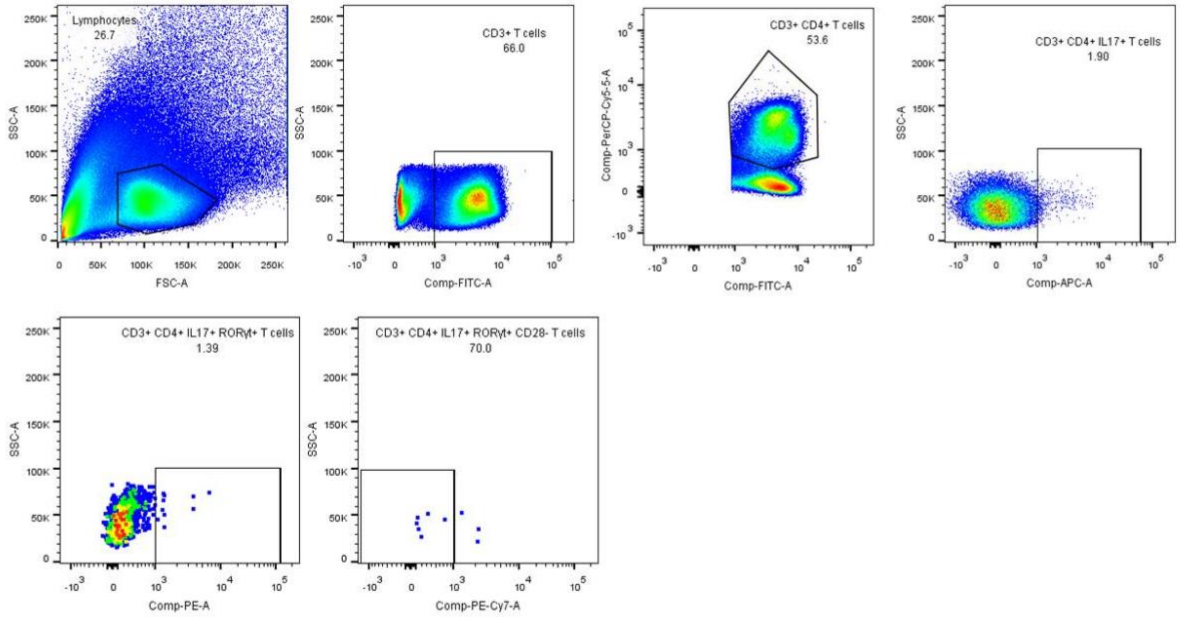

Supplementary Figure 1: Gating strategy for Th17 cell population ($CD3^+CD4^+IL17^+ROR\gamma t^+$ T cells) and aged Th17 cells ($CD3^+CD4^+IL17^+ROR\gamma t^+CD28^-$ T cells).

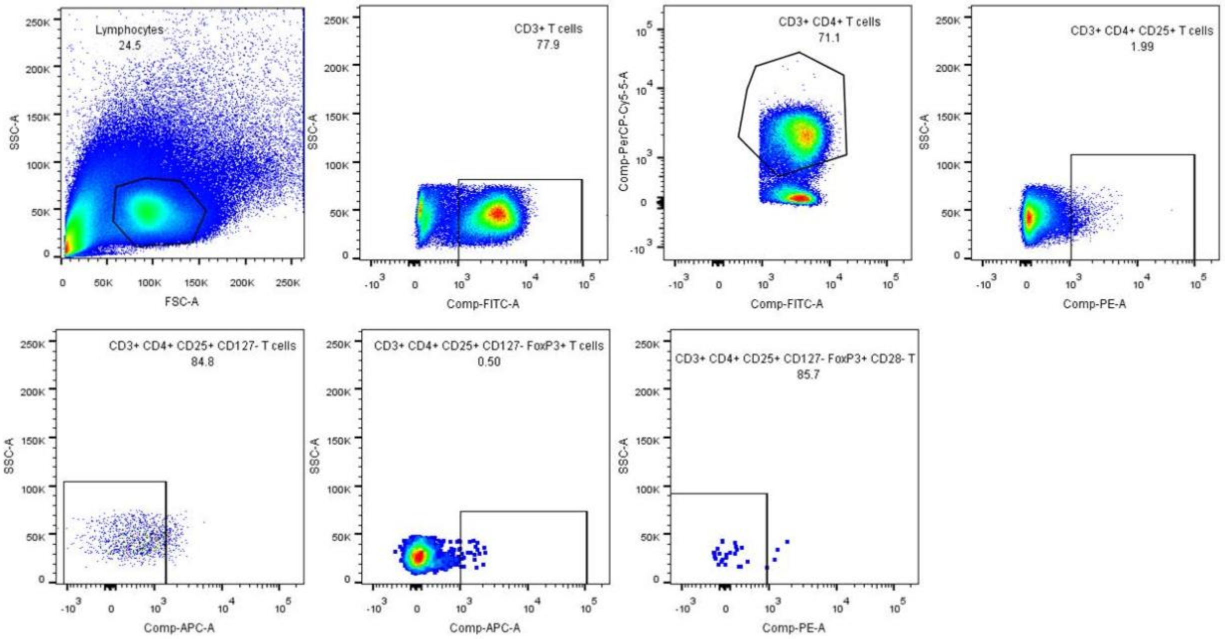

Supplementary Figure 2: Gating strategy for Treg cell population (CD3$^+$CD4$^+$CD25$^+$CD127$^-$ Foxp3$^+$ T cells) and aged Treg cells (CD3$^+$CD4$^+$CD25$^+$CD127$^-$Foxp3$^+$CD28$^-$ T cells).

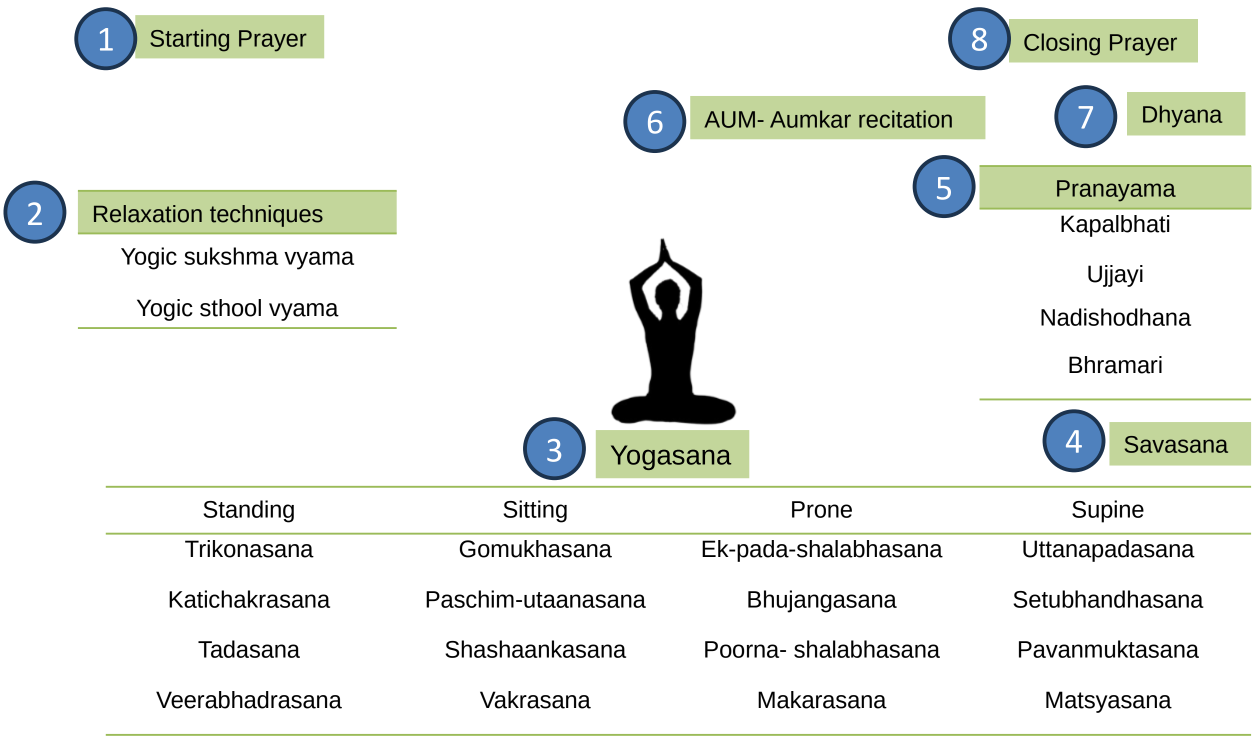

Supplementary Figure 3: Details of yogic activities performed in a single session of yoga program.
